# Exercise modalities associated with reduced functional disability in nonspecific neck pain: a network meta-analysis and exploratory dose-response analysis

**DOI:** 10.7717/peerj.21534

**Published:** 2026-07-23

**Authors:** Jindong Guo, Jingyi Xie, Xiang Li

**Affiliations:** 1 School of Sports Economics and Management, Hubei University of Economics, Wuhan, China; 2 School of Physical Education, Central China Normal University, Wuhan, China; 3 Physical Education Department, Huazhong Agricultural University, Wuhan, China

**Keywords:** Exercise, Neck pain, Network meta-analysis

## Abstract

**Background:**

Nonspecific neck pain (NSNP) is a common musculoskeletal condition that often leads to substantial functional disability and reduced quality of life. Exercise therapy is central to its management. However, the comparative effectiveness of specific modalities and dose-related patterns remains unclear.

**Methods:**

We conducted a systematic review and network meta-analysis (NMA) including 51 randomized controlled trials with 4,140 participants. Functional disability, measured using validated instruments, was the primary outcome. A Bayesian random-effects NMA was performed to compare exercise modalities, and a dose-response analysis examined the association between exercise parameters and outcomes. Effects were summarized as standardized mean differences (SMDs) with 95% credible intervals (CrIs).

**Results:**

Mind-body exercise was associated with a greater reduction in NSNP-related functional disability than control (SMD −0.363; 95% CrI [−0.516 to −0.151]). Resistance training (SMD −0.359; 95% CrI [−0.611 to −0.102]), flexibility training (SMD −0.352; 95% CrI [−0.623 to −0.078]), and coordination training (SMD −0.328; 95% CrI [−0.579 to −0.076]) were also associated with reductions in NSNP-related functional disability relative to control. Exploratory dose-response analysis suggested a possible non-linear association between per-session exercise dose, expressed pragmatically as MET-min, and NSNP-related functional disability. Estimated reductions were observed from around 100 MET-min per session, with larger study-level effects around 300–400 MET-min per session.

**Conclusion:**

Mind-body exercise, resistance training, flexibility training, and coordination training appear to be relatively favorable modalities for reducing NSNP-related functional disability. The dose-response findings should be interpreted cautiously, given the heterogeneity of the interventions and the limitations of the available study-level data.

## Introduction

Nonspecific neck pain (NSNP) is a common musculoskeletal condition with a substantial global burden (Anonymous, 2018). It refers to pain in the cervical region without an identifiable pathological cause, such as trauma, tumor, infection, or systemic disease. In routine clinical settings, patients with chronic or recurrent NSNP frequently report functional disability that interferes with daily life. In this context, functional disability denotes limitations in activities of daily living, the ability to meet occupational demands, and participation in social roles attributable to NSNP (Jahre et al., 2020). Typical difficulties include maintaining sustained postures required for reading and computer work, performing tasks that depend on cervical mobility, such as driving, and coping with a general reduction in physical capacity (Gaban et al., 2025). These restrictions accumulate over time and often lead to reduced quality of life and measurable socioeconomic costs for individuals and health systems. Reducing NSNP-related disability is therefore a central aim of conservative care.

Exercise therapy is widely used in the conservative management of NSNP. As an active, nonpharmacological approach, a structured program can generate physiological and functional benefits relevant to the core impairments of this condition (Gordon & Bloxham, 2016). Exercise may also promote local tissue metabolism and improve overall physical conditioning. Beyond these physiological effects, engaging patients in an active plan of care encourages self-management and may disrupt the reinforcing cycle of pain, fear-avoidance behaviors, and deconditioning that often sustains functional decline (Aguayo-Alves et al., 2024). In practice, clinicians draw on multiple exercise modalities; however, treatment choices are frequently guided by local preference and patient tolerance rather than comparative evidence.

Although exercise is commonly recommended for NSNP, uncertainty remains regarding which exercise modalities are associated with greater reductions in NSNP-related functional disability and how dose-related patterns relate to disability outcomes. Evidence regarding dose-related patterns, particularly per-session exercise dose and weekly exercise frequency, remains limited with respect to disability outcomes. Clarifying these issues may help clinicians tailor exercise prescriptions to functional goals while maintaining feasibility in routine practice. To address these gaps, we conducted a network meta-analysis (NMA) to compare exercise modalities and performed a dose–response analysis to examine how exercise dose and frequency relate to functional disability.

## Methods

### Protocol and registration

This systematic review and network meta-analysis were prospectively registered (PROSPERO; registration number CRD420251146651). The study was designed and conducted in accordance with the Preferred Reporting Items for Systematic Reviews and Meta-Analyses 2020 statement (PRISMA 2020) and the PRISMA extension for Network Meta-Analyses (PRISMA-NMA) (Cumpston et al., 2019; Hutton et al., 2015; Page et al., 2021). As the research synthesized data from previously published studies, ethical approval and informed consent were not required. A preliminary exploratory search was conducted between September 1, 2025, and September 10, 2025, to refine the scope of the review, finalize the eligibility criteria, and confirm the feasibility of the research question before registration. Formal systematic screening based on the finalized criteria began only after the protocol had been submitted to PROSPERO.

### Search strategy and study selection

A comprehensive search was initially performed in PubMed, Embase, Web of Science, and the Cochrane Library from database inception to July 2025. An updated supplementary search was performed on May 20, 2026, using the same databases and search strategies to identify additional eligible studies published up to that date. The search strategy combined Medical Subject Headings and free-text terms related to nonspecific neck pain, exercise therapy, physical activity, and specific modalities such as aerobic training, resistance training, flexibility training, and coordination training. The search was restricted to publications in English. Reference lists of relevant systematic reviews and meta-analyses were also screened to identify additional eligible studies (Appendix 1). Two reviewers (JG and JX) independently screened titles and abstracts according to the predefined criteria. Full-text articles deemed potentially eligible were subsequently assessed in detail. Disagreements were resolved through discussion and, when necessary, consultation with a third senior reviewer (XL).

### Eligibility criteria

We applied the Participants, Interventions, Comparators, Outcomes, and Study Design framework.

Participants. Adults aged 18 years or older with a clinical diagnosis of NSNP, defined as neck pain of mechanical origin, of any duration, without signs of a specific underlying pathology.

Interventions. Any structured exercise program delivered for a specified duration. Interventions included resistance training, flexibility training, mind-body exercise, combined resistance and coordination training, aerobic training, coordination training, rehabilitation training, combined aerobic and resistance training (Appendix 2).

Comparators. Eligible trials included either (1) one or more active exercise interventions *versus* a non-exercise control group (*e.g.*, usual care, waitlist, placebo) or (2) a head-to-head comparison between two or more active exercise interventions.

Outcomes. The primary outcome was change in NSNP-related functional disability measured with a validated patient-reported instrument, including the Neck Disability Index, Northwick Park Neck Pain Questionnaire, or other validated scales of neck-related functional status.

Study design. Only randomized controlled trials (RCTs) were included.

### Exclusion criteria

We excluded studies involving neck pain attributable to specific pathologies, such as cervical radiculopathy, myelopathy, fracture, infection, inflammatory diseases including rheumatoid arthritis, or malignancy. We also excluded case reports, observational studies, conference abstracts, and systematic reviews. Studies that did not provide sufficient data to calculate effect sizes were excluded if the required data could not be obtained from the authors.

### Data extraction and analysis

All statistical procedures were conducted in R version 4.3.2. We first synthesized evidence on the comparative effectiveness of exercise modalities and then examined the dose–response relationship.

### Data extraction and coding

Initial data were extracted between September 14, 2025, and September 30, 2025, by two reviewers (JG and JX) working independently using a standardized spreadsheet. An updated supplementary search was performed on May 20, 2026, using the same databases and search strategies to identify additional eligible studies published after the original search. Data from the newly included studies were extracted and checked by the same two reviewers using the same standardized procedures.

We recorded study characteristics, including first author and publication year, and participant demographics, including sample size, age, sex, and duration of NSNP symptoms. We also extracted intervention details, including exercise type, session duration, exercise frequency, and per-session exercise dose, as well as outcome data for NSNP-related functional disability, including means, standard deviations, and sample sizes at baseline and postintervention (Wan et al., 2014). When studies reported other measures of variance, these were converted to standard deviations. To ensure all data were on a comparable scale for analysis, measures of variance were consistently converted to standard deviations (SD). When studies reported standard error (SE), the SD was calculated using the standard formula: $\mathrm{SD}=\mathrm{SE}\times \sqrt{\mathrm{n}}$, where n represents the group’s sample size.

For the dose–response analysis, we pragmatically quantified per-session exercise dose using metabolic equivalent of task minutes per session (MET-min), calculated by multiplying the assigned MET value of the activity by session duration in minutes. For example, a 40-minute session of yoga (MET value ≈ 2.5) corresponds to 100 MET-min, whereas a 60-minute session of moderate resistance training (MET value ≈ 5.0) corresponds to 300 MET-min. MET values were assigned according to the 2024 Compendium of Physical Activities and the American College of Sports Medicine guidelines (Page et al., 2021; American College of Sports Medicine, 2021). This study-level harmonization metric was used to enable quantitative comparison across heterogeneous interventions. However, equivalent MET-min values across modalities should not be interpreted as equivalent physiological or therapeutic dose, particularly for mind-body and coordination-based interventions (Xie, Jin & Wang, 2025).

### Risk of bias and certainty of evidence assessment

Two reviewers (JG and JX) independently assessed the risk of bias for each RCT using the revised Cochrane Risk of Bias 2 tool (Sterne et al., 2019). Discrepancies were resolved through consultation with a third reviewer. We evaluated the certainty of the evidence for the primary outcome using the Grading of Recommendations, Assessment, Development and Evaluation (GRADE) framework, considering risk of bias, inconsistency, indirectness, imprecision, and publication bias (Guyatt et al., 2008).

### Network meta-analysis

We performed a Bayesian random-effects NMA using the gemtc package in R to synthesize direct and indirect evidence across all included modalities (Van Valkenhoef et al., 2012). Non-exercise control conditions, such as usual care and health education, were pooled into a single Control node to create a connected network. To synthesize data from the various validated scales used to measure neck-related disability (*e.g.*, Neck Disability Index, Copenhagen Neck Functional Disability Scale), we selected the standardized mean difference (SMD) as the summary effect measure, reported with 95% credible intervals (CrIs). The SMD converts outcomes from different instruments into a common, unitless metric, thereby enabling their valid comparison and pooling in the meta-analysis. We ranked interventions using the Surface Under the Cumulative Ranking Curve, in which higher values indicate a greater probability of being among the most effective options (Curteis et al., 2025).

### Dose–response analysis

We conducted a Bayesian dose–response meta-analysis using the dosresmeta package to evaluate the association between exercise dose in MET-min and changes in NSNP-related functional disability (Shim & Lee, 2019). We compared functional forms, including linear, quadratic, and restricted cubic spline models, and selected the best-fitting model using the Deviance Information Criterion. Distribution-based thresholds on the standardized effect scale were derived from the observed distribution of SMD effect sizes. Specifically, for each outcome *o*, a threshold was calculated as ${\mathrm{T}}_{\mathrm{o}}={\overline{\mathrm{SMD}}}_{\mathrm{o}}-\lambda \times \mathrm{SD}(\mathrm{SM}{\mathrm{D}}_{\mathrm{o}})$, where ${\overline{\mathrm{SMD}}}_{\mathrm{o}}$ denotes the mean of the observed SMD values for outcome *o,* and *SD* (SMD_o_) denotes their standard deviation. Outcome-specific thresholds were then pooled using study-count weighting. The scaling constant *λ* was calibrated such that the pooled threshold corresponded to −0.20 on the standardized effect scale. This value was used as a descriptive benchmark on the standardized effect scale to aid interpretation of the dose–response models, without implying a minimum clinically important difference or establishing clinical significance in original outcome units.

### Assessment of model assumptions and robustness

We quantified statistical heterogeneity across the network using the between-study standard deviation *τ* (Dias et al., 2014). The assumption of consistency was assessed by comparing the DIC values of the consistency and inconsistency models for a global test and by applying node-splitting for local tests. For node-splitting analyses, a Bayesian *p*-value greater than 0.05 was interpreted as indicating no strong evidence of local inconsistency (Dias et al., 2010). Potential publication bias was evaluated visually using comparison-adjusted funnel plots and formally using Egger’s regression test (Chaimani et al., 2013). To explore sources of heterogeneity, we planned network meta-regression analyses on prespecified moderators, including baseline pain severity, symptom duration, and participant age. Robustness was examined through two sensitivity analyses.

## Results

### Study selection

**Figure 1:**
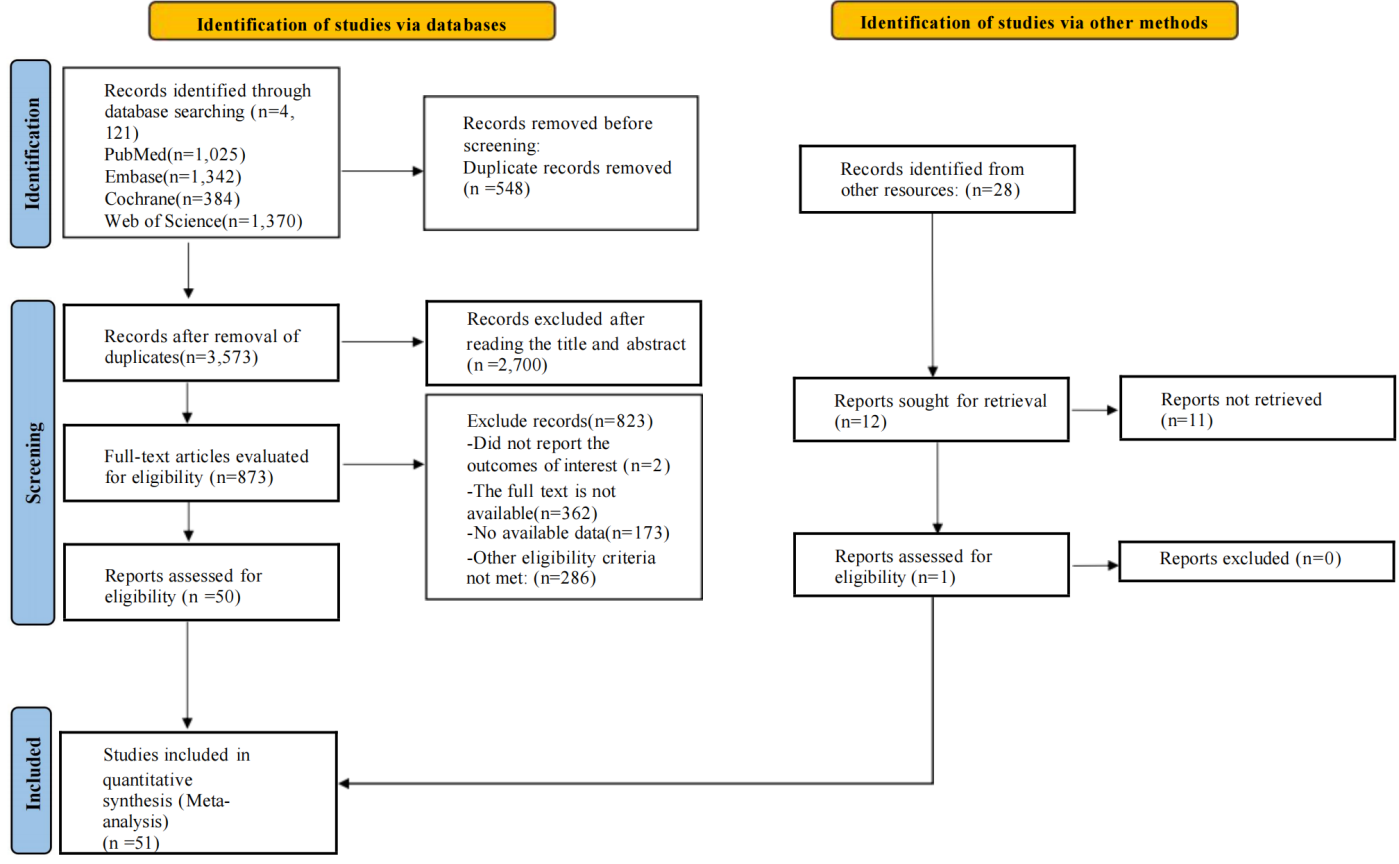
Literature review flowchart.

After duplicates were removed, 4,121 unique records were screened, and 873 articles underwent full-text assessment. In total, 51 RCTs were included. Across these trials, 4,140 participants were enrolled, of whom 3,085 (74.52%) were women (Fig. 1). The frequency of exercise interventions ranged from one to seven sessions per week. Interventions were categorized as rehabilitation training (10 studies), combined resistance and coordination training (one study), mind-body exercise (14 studies), combined aerobic and resistance training (four studies), coordination training (three studies), aerobic training (eight studies), flexibility training (23 studies), and resistance training (seven studies) (Appendix 3). These category counts were based on exercise intervention arms rather than mutually exclusive trials; therefore, trials comparing two or more exercise modalities could contribute to more than one category.

### Risk of bias and certainty of evidence

For the randomization process, 18 studies were rated as low risk, 27 had some concerns due to insufficient reporting, and six were rated as high risk. The certainty of the evidence for the main NMA comparisons on NSNP-related functional disability, assessed using the GRADE framework, ranged from very low to moderate. Downgrading was driven primarily by risk of bias in the included studies, inconsistency across trials, and imprecision in the effect estimates (Appendix 4, Appendix 5).

### NMA results

**Figure 2:**
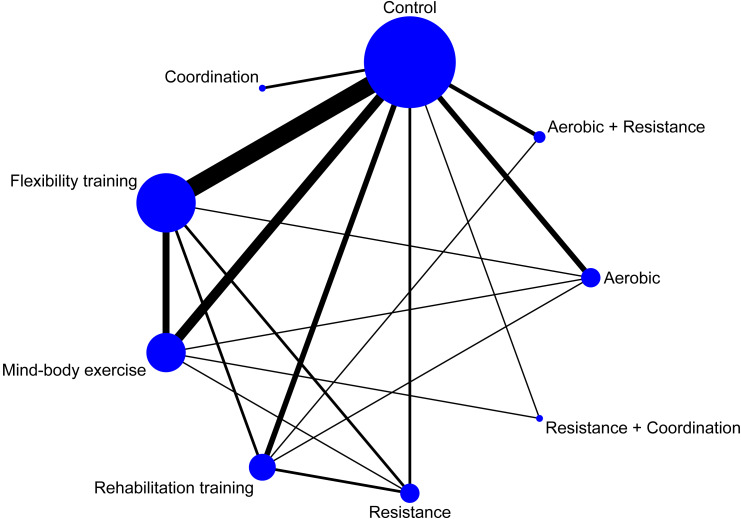
Network diagrams.

We evaluated the network using standard diagnostics, including consistency checks (Fig. 2). No strong evidence of inconsistency between direct and indirect evidence was identified based on node-splitting analyses (Appendix 6). Overall heterogeneity was moderate, based on the posterior distribution of the between-study standard deviation (*τ*), supporting the use of a random-effects model.

Compared with control, mind-body exercise showed the largest estimated reduction in NSNP-related functional disability (SMD −0.363; 95% CrI [−0.516 to −0.151]), followed by resistance training (SMD −0.359; 95% CrI [−0.611 to −0.102]), flexibility training (SMD −0.352; 95% CrI [−0.623 to −0.078]), and coordination training (SMD −0.328; 95% CrI [−0.579 to −0.076]). These interventions were associated with greater reductions in NSNP-related functional disability than control.

**Figure 3:**
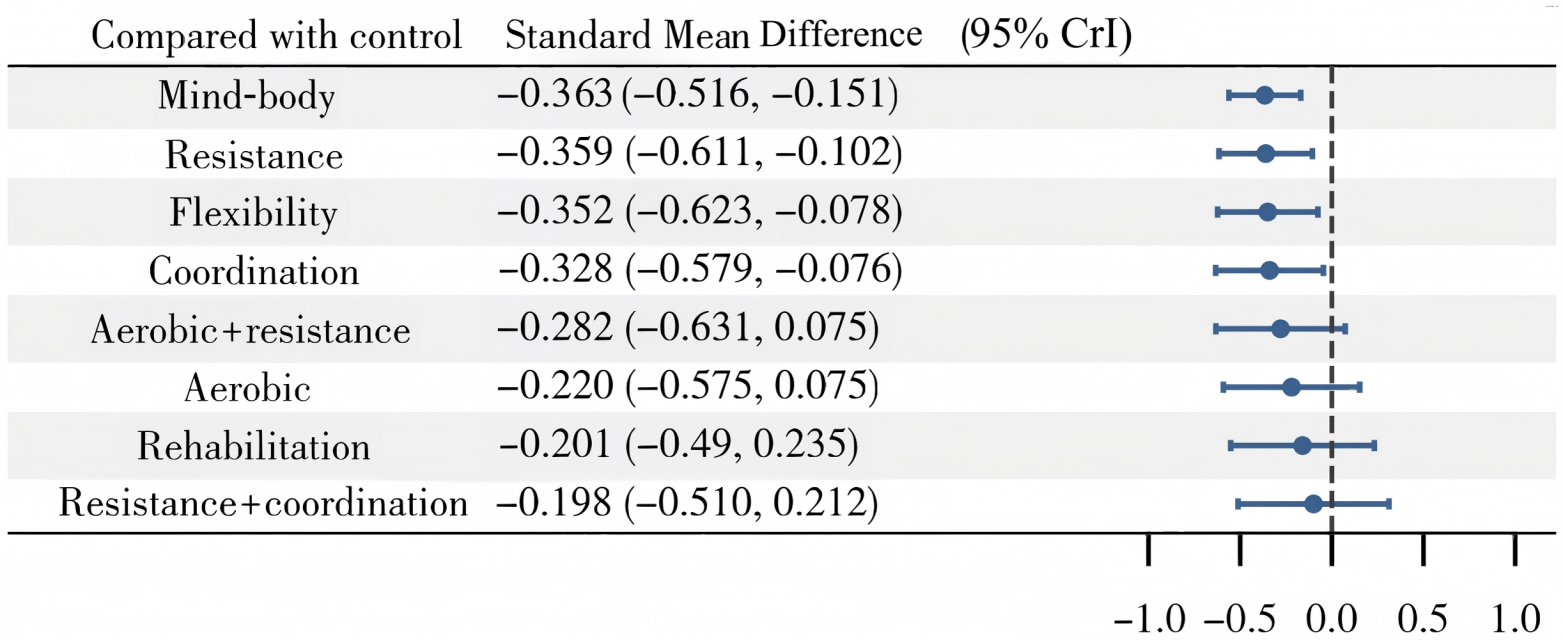
Network meta-analysis forest plot of various exercise interventions for NSNP-related functional disability.

The league table summarizes all pairwise comparisons based on the combined direct and indirect evidence. Mind-body exercise appeared to be associated with a greater reduction in NSNP-related functional disability than resistance training (SMD −0.26; 95% CrI [−0.30 to −0.22]). Flexibility training did not show a clear difference from resistance training (SMD −0.29; 95% CrI [−0.98 to 0.39]), but was associated with a greater reduction in NSNP-related functional disability than coordination training (SMD −0.52; 95% CrI [−0.91 to −0.18]). Aerobic training was associated with a greater reduction in NSNP-related functional disability than rehabilitation training (SMD −0.22; 95% CrI [−0.42 to −0.02]) (Fig. 3, Appendix 7). The comparison-adjusted funnel plot appeared approximately symmetrical, suggesting no clear evidence of important publication bias (Appendix 8).

### Dose–response analysis

**Figure 4:**
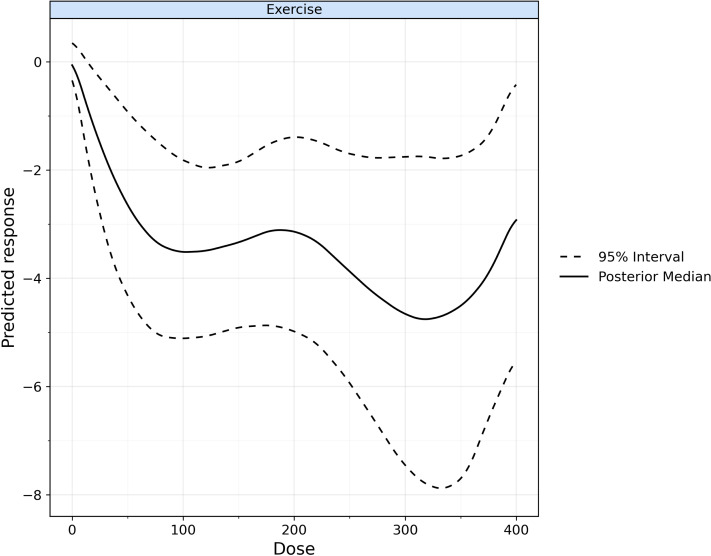
Dose–response association between per-session exercise dose and NSNP-related functional disability.

A possible non-linear association was identified between exercise dose and NSNP-related functional disability. Based on the model estimates, reductions in NSNP-related functional disability appeared to emerge at around 100 MET-min per session. Larger study-level effects were observed around 300–400 MET-min per session, after which the curve appeared to plateau. For weekly exercise frequency, reductions in NSNP-related functional disability appeared to emerge from approximately two sessions per week, with larger study-level effects observed at five to six sessions per week (Fig. 4, Appendix 9).

### Network meta-regression

Network meta-regression was conducted to examine potential moderators. None of the variables tested, including participant age, intervention duration, frequency, publication year, study population, or intervention timing, showed a statistically significant influence on the relative treatment effects for NSNP-related functional disability (Appendix 10).

## Discussion

### Principal findings

The NMA suggested that mind-body exercise, resistance training, flexibility training, and coordination training were associated with reductions in NSNP-related functional disability compared with control. However, clinical interpretation should consider heterogeneity and the certainty of the evidence. The dose–response analysis suggested a possible non-linear, plateauing pattern. In the study-level analysis, reductions in NSNP-related functional disability appeared to emerge at around 100 MET-min per session, while larger study-level effects were observed around 300–400 MET-min per session. A similar pattern was observed for weekly exercise frequency, with reductions in NSNP-related functional disability appearing to emerge from approximately two sessions per week. These findings should be interpreted cautiously and regarded as hypothesis-generating rather than prescriptive.

### Comparison with existing evidence

The findings suggest an apparent relative ordering of exercise modalities with respect to NSNP-related functional disability. Prior literature has emphasized the importance of neuromuscular control, proprioception, and cervical range of motion in NSNP rehabilitation (Chaimani et al., 2013). Mind-body exercise, such as yoga and pilates, had the highest ranking for NSNP-related functional disability in this analysis. This finding may reflect its multicomponent structure, which includes physical postures, motor control, interoceptive awareness, and breathing techniques (Blanpied et al., 2017; Gard et al., 2014; Villemure et al., 2015), although the relative contribution of each component could not be determined from the available study-level data. Resistance training ranked second in this review, which is consistent with the potential role of strengthening, particularly of the deep cervical flexors and scapular stabilizers, in rehabilitation (O’Leary et al., 2009; Saragiotto et al., 2016). The ranking of mind-body exercise may also suggest that multicomponent interventions incorporating strengthening, motor control, and proprioceptive elements warrant further study (Van Dieën et al., 2019). However, this inference remains speculative. Flexibility and coordination training also showed favorable rankings. These findings are consistent with the relevance of cervical range of motion, muscle stiffness, proprioception, and neuromuscular control in NSNP rehabilitation, but they should not be interpreted as direct evidence of specific mechanisms (Tunwattanapong, Kongkasuwan & Kuptniratsaikul, 2016; Röijezon et al., 2008).

General physical activity recommendations, such as 150 min of moderate-intensity activity per week, are not specific to NSNP rehabilitation (WHO Guidelines Approved by the Guidelines Review Committee, 2020). In this study-level synthesis, estimated reductions in NSNP-related functional disability appeared to emerge at around 100 MET-min per session, with larger study-level effects observed around 300–400 MET-min per session and at moderate-to-higher weekly exercise frequencies. However, these observations are descriptive, may differ across exercise modalities, and should not be interpreted as modality-invariant prescription targets (Nijs et al., 2012). Larger exercise doses should not be assumed to produce proportionally greater benefit, and further primary studies are needed to clarify modality-specific dose–response relationships.

### Clinical implications

These findings may inform exercise-based management of NSNP-related functional disability in several ways. First, clinicians may consider modalities such as mind-body exercise, resistance training, flexibility training, and coordination training, as these were associated with greater reductions in NSNP-related functional disability than control. Second, the exploratory dose–response analysis may provide contextual information for clinical exercise planning. Across the included trials, estimated reductions in NSNP-related functional disability appeared to emerge at around 100 MET-min per session and approximately two sessions per week, while larger study-level effects were observed around 300–400 MET-min per session. These observed values may help inform broad considerations about exercise initiation and progression, but they should be interpreted as approximate study-level reference points rather than as minimum effective doses, optimal targets, or modality-invariant recommendations. Third, treatment planning should remain patient-centered. Group-level estimates guide decision-making; however, clinicians should integrate the patient’s specific functional limitations, preferences, and capacity to design a safe, sustainable, and engaging program that targets individualized goals for functional recovery.

### Strengths and limitations

This study has several strengths. First, the NMA allowed the simultaneous comparison of multiple exercise modalities and provided a probabilistic ranking of their relative effectiveness in reducing NSNP-related functional disability, thereby offering comparative evidence across interventions. Second, in addition to comparing exercise modalities, the study explored dose–response patterns for NSNP-related functional disability outcomes, thereby providing additional insight into how exercise dose may relate to this primary outcome.

These findings should be interpreted in light of several limitations. First, the conclusions depend on the quality and reporting of the included trials, and undetected risk of bias cannot be excluded. Second, substantial clinical heterogeneity is inherent in exercise research. Categories such as mind-body exercise or coordination training represent broad classes rather than homogeneous intervention entities and likely varied in content, supervision, duration, and intensity across studies. This within-category variability may have introduced residual heterogeneity and some misclassification. Third, MET-min was used as a pragmatic study-level exercise dose metric, but it may not adequately capture the qualitative, coordinative, or behavioral components of all exercise modalities. Equivalent MET-min values across modalities should therefore not be interpreted as equivalent physiological load or therapeutic exercise dose. Accordingly, the dose–response findings should be interpreted cautiously as exploratory, study-level observations rather than as clinically prescriptive thresholds. Fourth, women accounted for 74.52% of the included population; therefore, the generalizability of the findings to the broader NSNP population, particularly to men, should be interpreted with caution. Fifth, age-specific outcome structures could not be fully examined because most included studies did not report disability outcomes stratified by age. Since aging may influence exercise capacity, tolerance, and response to exercise interventions, clinicians should consider age-related factors when applying these findings in practice.

## Conclusion

This network meta-analysis compared exercise modalities and explored study-level dose–response patterns for NSNP-related functional disability. Mind-body exercise, resistance training, flexibility training, and coordination training were associated with greater reductions in NSNP-related functional disability than control and appeared relatively favorable within the current evidence base. Exploratory dose–response analyses suggested a possible non-linear association between MET-min per session and NSNP-related functional disability outcomes. However, these findings should be interpreted cautiously given the use of MET-min as a pragmatic exercise dose metric and the heterogeneity within intervention categories. The dose–response results should therefore be regarded as hypothesis-generating rather than prescriptive.

##  Supplemental Information

10.7717/peerj.21534/supp-1 Supplemental Information 1 Appendix

10.7717/peerj.21534/supp-2 Supplemental Information 2 Code

10.7717/peerj.21534/supp-3 Supplemental Information 3 Translation codebook

10.7717/peerj.21534/supp-4 Supplemental Information 4 PRISMA checklist

## Additional Information and Declarations

### Data Availability

The following information was supplied regarding data availability: This is a Systematic review/Meta-analysis.
