# Supplementary material for: Exercise modalities associated with reduced functional disability in nonspecific neck pain: a network meta-analysis and exploratory dose-response analysis
Source: PeerJ. 2026 Jul 23;14:e21534. doi: 10.7717/peerj.21534 (PMC13401843; doi:10.7717/peerj.21534)
Supplement: Supplemental Information 1 [file peerj-14-21534-s001.docx]

**Exercise modalities associated with reduced functional disability in nonspecific neck pain: A network meta-analysis and exploratory dose-response analysis**

**Supplementary Files**

**Contents**

| **Supplement 1: Search Strategy** |
| --- |
| **Supplement 2: Exercise categories** |
| **Supplement 3: Characteristics of Included Studies** |
| **Supplement 4: Risk of Bias in Individual Studies** |
| **Supplement 5: The details of GRADE** |
| **Supplement 6: Node-splitting results** |
| **Supplement 7: League table** |
| **Supplement 8: Funnel plot** |
| **Supplement 9: Dose-response relationship** |
| **Supplement 10: Network meta-regression** |

**Supplement 1: Search Strategy**

## 2.1 MEDLINE

| **Search** | **Query** |
| --- | --- |
| #1 | "Neck Pain"[Mesh] |
| #2 | (((((((((((((((((((((((((((((((((((Neck Pains) OR (Pain, Neck)) OR (Pains, Neck)) OR (Neckache)) OR (Neckaches)) OR (Cervical Pain)) OR (Cervical Pains)) OR (Pain, Cervical)) OR (Cervicalgia)) OR (Cervicalgias)) OR (Cervicodynia)) OR (Cervicodynias)) OR (Neck Ache)) OR (Ache, Neck)) OR (Aches, Neck)) OR (Anterior Cervical Pain)) OR (Anterior Cervical Pains)) OR (Cervical Pain, Anterior)) OR (Cervical Pains, Anterior)) OR (Pain, Anterior Cervical)) OR (Anterior Neck Pain)) OR (Neck Pain, Anterior)) OR (Neck Pains, Anterior)) OR (Pain, Anterior Neck)) OR (Pains, Anterior Neck)) OR (Posterior Cervical Pain)) OR (Cervical Pain, Posterior)) OR (Cervical Pains, Posterior)) OR (Pain, Posterior Cervical)) OR (Pains, Posterior Cervical)) OR (Posterior Cervical Pains)) OR (Neck Pain, Posterior)) OR (Neck Pains, Posterior)) OR (Pain, Posterior Neck)) OR (Pains, Posterior Neck)) OR (Posterior Neck Pains) |
| #3 | ("Neck Pain"[Mesh]) OR ((((((((((((((((((((((((((((((((((((Neck Pains) OR (Pain, Neck)) OR (Pains, Neck)) OR (Neckache)) OR (Neckaches)) OR (Cervical Pain)) OR (Cervical Pains)) OR (Pain, Cervical)) OR (Cervicalgia)) OR (Cervicalgias)) OR (Cervicodynia)) OR (Cervicodynias)) OR (Neck Ache)) OR (Ache, Neck)) OR (Aches, Neck)) OR (Anterior Cervical Pain)) OR (Anterior Cervical Pains)) OR (Cervical Pain, Anterior)) OR (Cervical Pains, Anterior)) OR (Pain, Anterior Cervical)) OR (Anterior Neck Pain)) OR (Neck Pain, Anterior)) OR (Neck Pains, Anterior)) OR (Pain, Anterior Neck)) OR (Pains, Anterior Neck)) OR (Posterior Cervical Pain)) OR (Cervical Pain, Posterior)) OR (Cervical Pains, Posterior)) OR (Pain, Posterior Cervical)) OR (Pains, Posterior Cervical)) OR (Posterior Cervical Pains)) OR (Neck Pain, Posterior)) OR (Neck Pains, Posterior)) OR (Pain, Posterior Neck)) OR (Pains, Posterior Neck)) OR (Posterior Neck Pains)) |
| #4 | "Exercise"[Mesh] |
| #5 | (((((((((((((((((((((((((((((((Exercises) OR (Exercise, Physical)) OR (Exercises, Physical)) OR (Physical Exercise)) OR (Physical Exercises)) OR (Exercise, Aerobic)) OR (Aerobic Exercise)) OR (Aerobic Exercises)) OR (Exercises, Aerobic)) OR (Exercise, Isometric)) OR (Exercises, Isometric)) OR (Isometric Exercises)) OR (Isometric Exercise)) OR (Acute Exercise)) OR (Acute Exercises)) OR (Exercise, Acute)) OR (Exercises, Acute)) OR (Exercise Training)) OR (Exercise Trainings)) OR (Training, Exercise)) OR (Trainings, Exercise)) OR (Physical Activity)) OR (Activities, Physical)) OR (Activity, Physical)) OR (Physical Activities)) OR (Yoga)) OR (Pilates)) OR (Aquatic exercise)) OR (Qi gong)) OR (Wu qin xi)) OR (Stretch)) OR (Strength) Sort by: Most Recent |
| #6 | ("Exercise"[Mesh]) OR ((((((((((((((((((((((((((((((((Exercises) OR (Exercise, Physical)) OR (Exercises, Physical)) OR (Physical Exercise)) OR (Physical Exercises)) OR (Exercise, Aerobic)) OR (Aerobic Exercise)) OR (Aerobic Exercises)) OR (Exercises, Aerobic)) OR (Exercise, Isometric)) OR (Exercises, Isometric)) OR (Isometric Exercises)) OR (Isometric Exercise)) OR (Acute Exercise)) OR (Acute Exercises)) OR (Exercise, Acute)) OR (Exercises, Acute)) OR (Exercise Training)) OR (Exercise Trainings)) OR (Training, Exercise)) OR (Trainings, Exercise)) OR (Physical Activity)) OR (Activities, Physical)) OR (Activity, Physical)) OR (Physical Activities)) OR (Yoga)) OR (Pilates)) OR (Aquatic exercise)) OR (Qi gong)) OR (Wu qin xi)) OR (Stretch)) OR (Strength)) |
| #7 | ((Randomized controlled trial[Title/Abstract]) OR (Randomized[Title/Abstract])) OR (Placebo[Title/Abstract]) |
| #9 | #3 AND #6 AND #7 AND #7 |

**Supplementary 2: Exercise categories**

Table S1 Exercise categories

| Category | Definition |
| --- | --- |
| **Aerobic Exercise Training** | Activities involving large muscle groups, performed rhythmically for a sustained period (typically >10 min), primarily aimed at improving cardiorespiratory endurance. (1). |
| **Resistance Training** | Exercises involving muscle contractions against external resistance (e.g., weights, bands, bodyweight, machines) aimed at increasing muscular strength, power, endurance, or mass(2). |
| **Flexibility Training** | Exercises involving stretching muscles and connective tissues through specific movements or postures (static or dynamic) aimed at improving or maintaining range of motion (ROM)(3). |
| **Coordination Training** | Exercises designed to improve the ability of different body parts to work together smoothly and efficiently, involving proprioception, reaction time, agility, and motor control (4). |
| **Mind-Body training** | Practices combining physical postures/movements, breathing control, and meditation/mindfulness/concentration, aimed at improving both physical and mental well-being. Examples: Tai Chi, Yoga, Qigong (5). |
| **Combined Aerobic and Resistance Training** | Interventions explicitly reported to include structured components of both aerobic exercise **and** resistance training within the program, often with defined protocols for each (6). |
| **Combined Resistance and Coordination Training** | Interventions primarily focused on resistance training but **integrating** specific exercises aimed at improving balance, proprioception, or motor control as part of the regimen(7) |
| **Rehabilitation Training** | Functional training programs typically designed for recovery from specific injuries, diseases, or surgeries. May combine elements from various categories above, but the **primary goal is restoring specific functional deficits or addressing impairments**, rather than general fitness enhancement (8). |

Reference:

1. Thompson PD, Arena R, Riebe D, Pescatello LS. ACSM's new preparticipation health screening recommendations from ACSM's guidelines for exercise testing and prescription, ninth edition. Curr Sports Med Rep. 2013;12(4):215-7.

2. Kraemer WJ, Ratamess NA. Fundamentals of resistance training: progression and exercise prescription. Med Sci Sports Exerc. 2004;36(4):674-88.

3. Vergeer I, Roberts J. Movement and stretching imagery during flexibility training. J Sports Sci. 2006;24(2):197-208.

4. Garber CE, Blissmer B, Deschenes MR, Franklin BA, Lamonte MJ, Lee IM, et al. American College of Sports Medicine position stand. Quantity and quality of exercise for developing and maintaining cardiorespiratory, musculoskeletal, and neuromotor fitness in apparently healthy adults: guidance for prescribing exercise. Med Sci Sports Exerc. 2011;43(7):1334-59.

5. Fernández-Rodríguez R, Álvarez-Bueno C, Cavero-Redondo I, Torres-Costoso A, Pozuelo-Carrascosa DP, Reina-Gutiérrez S, et al. Best Exercise Options for Reducing Pain and Disability in Adults With Chronic Low Back Pain: Pilates, Strength, Core-Based, and Mind-Body. A Network Meta-analysis. J Orthop Sports Phys Ther. 2022;52(8):505-21.

6. Liang Z, Zhang M, Wang C, Hao F, Yu Y, Tian S, et al. The Best Exercise Modality and Dose to Reduce Glycosylated Hemoglobin in Patients with Type 2 Diabetes: A Systematic Review with Pairwise, Network, and Dose-Response Meta-Analyses. Sports Med. 2024;54(10):2557-70.

7. Granacher U, Behm DG. Relevance and Effectiveness of Combined Resistance and Balance Training to Improve Balance and Muscular Fitness in Healthy Youth and Youth Athletes: A Scoping Review. Sports Med. 2023;53(2):349-70.

8. Lee KE, Choi M, Jeoung B. Effectiveness of Rehabilitation Exercise in Improving Physical Function of Stroke Patients: A Systematic Review. Int J Environ Res Public Health. 2022;19(19).

**Supplement 3. Characteristics of Included Studies**

| **cov_id** | **studyID** | **country** | **funded** | **trt** | **outcome** | **intensity_met** | **sessions** | **weeks** | **pc_female** |
| --- | --- | --- | --- | --- | --- | --- | --- | --- | --- |
| 1 | Yun2015 | Korea | N | Aerobic | NDI | 4.5 | 3 | 4 | 0.8 |
| 1 | Yun2015 | Korea | N | Control | NDI | NA | NA | NA | 0.8 |
| 2 | Michalsen2012 | Germany | N | Flexibility training | NPAD | 3 | 3 | 4 | 0.82 |
| 2 | Michalsen2012 | Germany | N | Mind-body exercise | NPAD | 5.5 | 1 | 9 | 0.92 |
| 3 | Trott2009 | Germany | Y | Mind-body exercise | NPAD | 3.3 | 3 | 12 | 0.95 |
| 3 | Trott2009 | Germany | Y | Resistance + Coordination | NPAD | 5 | 3 | 12 | 0.95 |
| 3 | Trott2009 | Germany | Y | Control | NPAD | NA | NA | NA | 0.95 |
| 4 | Kang2021 | Korea | Y | Flexibility training | NDI | 3 | 3 | 6 | 0.62 |
| 4 | Kang2021 | Korea | Y | Control | NDI | NA | NA | NA | 0.56 |
| 5 | Tunwattanapong2016 | Thailand | Y | Flexibility training | NDI | 3 | 5 | 4 | 0.62 |
| 5 | Tunwattanapong2016 | Thailand | Y | Control | NDI | NA | NA | NA | 0.56 |
| 6 | Picak2023 | Turkey | N | Mind-body exercise | NDI | 6 | 2 | 6 | 0.87 |
| 6 | Picak2023 | Turkey | N | Control | NDI | NA | NA | NA | 0.92 |
| 7 | Ris2016 | Denmark | Y | Aerobic | NDI | 5.5 | 3 | 16 | 0.68 |
| 7 | Ris2016 | Denmark | Y | Control | NDI | NA | NA | NA | 0.8 |
| 8 | Rendant2011 | Germany | N | Mind-body exercise | NPAD | 5.5 | 1 | 12 | 0.85 |
| 8 | Rendant2011 | Germany | N | Flexibility training | NPAD | 3 | 1 | 12 | 0.89 |
| 8 | Rendant2011 | Germany | N | Control | NPAD | NA | NA | NA | 0.87 |
| 9 | Park2015 | Korea | Y | Rehabilitation training | NDI | 6 | 3 | 6 | 0.86 |
| 9 | Park2015 | Korea | Y | Control | NDI | NA | NA | NA | 0.75 |
| 10 | Nandini2018 | India | N | Mind-body exercise | NDI | 5.5 | 5 | 12 | 0.46 |
| 10 | Nandini2018 | India | N | Control | NDI | NA | NA | NA | 0.43 |
| 11 | Ma2011 | China | Y | Flexibility training | NDI | 3 | 2 | 6 | 0.26 |
| 11 | Ma2011 | China | Y | Control | NDI | NA | NA | NA | 0.4 |
| 12 | Lytras2020 | Greece | N | Aerobic + Resistance | NDI | 6 | 4 | 10 | 0.25 |
| 12 | Lytras2020 | Greece | N | Control | NDI | NA | NA | NA | 0.25 |
| 13 | Li2017 | China | Y | Resistance | NDI | 4 | 3 | 6 | 1 |
| 13 | Li2017 | China | Y | Control | NDI | NA | NA | NA | 1 |
| 14 | Letafatkar2019 | Iran | N | Aerobic | NDI | 5.5 | 3 | 8 | 1 |
| 14 | Letafatkar2019 | Iran | N | Control | NDI | NA | NA | NA | 1 |
| 15 | Lee2016 | Korea | N | Mind-body exercise | NDI | 2.8 | 3 | 10 | 1 |
| 15 | Lee2016 | Korea | N | Control | NDI | NA | NA | NA | 1 |
| 16 | Lansinger2007 | Sweden | N | Mind-body exercise | NDI | 3.3 | 2 | 12 | 0.73 |
| 16 | Lansinger2007 | Sweden | N | Aerobic | NDI | 4.5 | 3 | 12 | 0.67 |
| 17 | Kim2016 | Korea | N | Rehabilitation training | NDI | 6 | 2 | 4 | 0.57 |
| 17 | Kim2016 | Korea | N | Flexibility training | NDI | 3 | 3 | 4 | 0.5 |
| 18 | Khosrokiani2017 | Iran | N | Flexibility training | NDI | 3 | 3 | 24 | 1 |
| 18 | Khosrokiani2017 | Iran | N | Control | NDI | NA | NA | NA | 1 |
| 19 | Karlsson2014 | Sweden | Y | Resistance | NDI | 3.5 | 3 | 24 | 1 |
| 19 | Karlsson2014 | Sweden | Y | Flexibility training | NDI | 3 | 3 | 24 | 1 |
| 20 | Gupta2013 | India | N | Rehabilitation training | NDI | 6 | 3 | 4 | 0.4 |
| 20 | Gupta2013 | India | N | Resistance | NDI | 6 | 3 | 4 | 0.4 |
| 21 | Lee2016_2 | Korea | N | Rehabilitation training | NDI | 5 | 3 | 10 | NA |
| 21 | Lee2016_2 | Korea | N | Aerobic | NDI | 4.5 | 3 | 10 | NA |
| 22 | Javdaneh2020 | Iran | Y | Coordination | NDI | 6 | 3 | 6 | 0.45 |
| 22 | Javdaneh2020 | Iran | Y | Control | NDI | NA | NA | NA | 0.54 |
| 23 | Javdaneh2021 | Norway | N | Aerobic | NPAD | 4.5 | 3 | 6 | 0.43 |
| 23 | Javdaneh2021 | Norway | N | Control | NPAD | NA | NA | NA | 0.43 |
| 24 | Hakkinen2008 | Finland | Y | Flexibility training | NSPD | 3 | 3 | 54 | 0.9 |
| 24 | Hakkinen2008 | Finland | Y | Resistance | NSPD | 3.5 | 3 | 54 | 0.9 |
| 25 | Groisman2019 | Brazil | N | Flexibility training | NDI | 3 | 2 | 4 | 0.84 |
| 25 | Groisman2019 | Brazil | N | Control | NDI | NA | NA | NA | 0.93 |
| 26 | GRIFFITHS2009 | UK | Y | Flexibility training | NPDS | 3 | 4 | 6 | 0.7 |
| 26 | GRIFFITHS2009 | UK | Y | Rehabilitation training | NPDS | 6 | 4 | 6 | 0.54 |
| 27 | ULUG2018 | Turkey | N | Mind-body exercise | NDI | 3.3 | 5 | 6 | 0.85 |
| 27 | ULUG2018 | Turkey | N | Resistance | NDI | 6 | 5 | 6 | 0.85 |
| 28 | Ghaderi2016 | Iran | Y | Coordination | NDI | 3.5 | 3 | 12 | NA |
| 28 | Ghaderi2016 | Iran | Y | Control | NDI | NA | NA | NA | NA |
| 29 | Kjellman2002 | Sweden | Y | Aerobic + Resistance | NDI | 7 | 2 | 8 | 0.8 |
| 29 | Kjellman2002 | Sweden | Y | Control | NDI | NA | NA | NA | 0.76 |
| 30 | BoriSut2013 | Thailand | N | Aerobic + Resistance | NDI | 6 | 3 | 12 | 1 |
| 30 | BoriSut2013 | Thailand | N | Rehabilitation training | NDI | 6 | 3 | 12 | 1 |
| 30 | BoriSut2013 | Thailand | N | Control | NDI | NA | NA | NA | 1 |
| 31 | Chiu2005 | China | Y | Flexibility training | VPNS | 3 | 2 | 6 | 0.28 |
| 31 | Chiu2005 | China | Y | Control | VPNS | NA | NA | NA | 0.33 |
| 32 | Cramer2013 | Germany | N | Mind-body exercise | NDI | 5.5 | 1 | 9 | 0.84 |
| 32 | Cramer2013 | Germany | N | Flexibility training | NDI | 3 | 1 | 9 | 0.8 |
| 33 | Cazotti2018 | Brazil | N | Mind-body exercise | NDI | 5 | 2 | 6 | 0.18 |
| 33 | Cazotti2018 | Brazil | N | Control | NDI | NA | NA | NA | 0.25 |
| 34 | Dunleavy2015 | USA | N | Mind-body exercise | NDI | 5.5 | 1 | 9 | 0.84 |
| 34 | Dunleavy2015 | USA | N | Flexibility training | NDI | 5 | 1 | 9 | 0.8 |
| 35 | Dusunceli2009 | Turkey | N | Flexibility training | NDI | 3 | 5 | 3 | 0.6 |
| 35 | Dusunceli2009 | Turkey | N | Control | NDI | NA | NA | NA | 0.7 |
| 36 | Cheng2022 | China | Y | Mind-body exercise | NDI | 6 | 3 | 8 | 0.7 |
| 36 | Cheng2022 | China | Y | Control | NDI | NA | NA | NA | 0.64 |
| 37 | Falla2013 | Turkey | N | Rehabilitation training | NDI | 6 | 2 | 8 | 1 |
| 37 | Falla2013 | Turkey | N | Control | NDI | NA | NA | NA | 1 |
| 38 | Evans2012 | USA | Y | Flexibility training | NDI | 3 | 4 | 12 | 0.73 |
| 38 | Evans2012 | USA | Y | Aerobic | NDI | 3.5 | 4 | 12 | 0.71 |
| 39 | Bernal-Utrera2020 | Spain | N | Rehabilitation training | NDI | 6 | 3 | 3 | 0.78 |
| 39 | Bernal-Utrera2020 | Spain | N | Control | NDI | NA | NA | NA | 0.55 |
| 40 | Galindez-Ibarbengoetxea2017 | Spain | N | Flexibility training | NDI | 3 | 3 | 1 | 1 |
| 40 | Galindez-Ibarbengoetxea2017 | Spain | N | Control | NDI | NA | NA | NA | 1 |
| 41 | Bharti2024 | India | N | Rehabilitation training | NDI | 3.3 | 5 | 4 | 0.66 |
| 41 | Bharti2024 | India | N | Resistance | NDI | 6 | 5 | 4 | 0.73 |
| 42 | Kuptniratsaikula2023 | Thailand | N | Mind-body exercise | NDI | 3.3 | 7 | 4 | 0.81 |
| 42 | Kuptniratsaikula2023 | Thailand | N | Flexibility training | NDI | 3 | 6 | 4 | 0.88 |
| 43 | Ahi2023 | Turkey | N | Flexibility training | NDI | 3 | 5 | 3 | 0.25 |
| 43 | Ahi2023 | Turkey | N | Control | NDI | NA | NA | NA | 0.29 |
| 44 | Nejati2020 | Iran | N | Resistance | NPDS | 6 | 3 | 12 | 0.88 |
| 44 | Nejati2020 | Iran | N | Control | NPDS | NA | NA | NA | 0.76 |
| 45 | Ei-gendy2019 | Egypt | N | Flexibility training | NDI | 3 | 3 | 4 | NA |
| 45 | Ei-gendy2019 | Egypt | N | Control | NDI | NA | NA | NA | NA |
| 46 | Khan2022 | Pakistan | N | Flexibility training | NDI | 3 | 3 | 2 | 0.5 |
| 46 | Khan2022 | Pakistan | N | Control | NDI | NA | NA | NA | 0.5 |
| 47 | Kashyap2018 | Saudi Arabia | N | Flexibility training | NDI | 3 | 3 | 1 | 1 |
| 47 | Kashyap2018 | Saudi Arabia | N | Control | NDI | NA | NA | NA | 1 |
| 48 | Phadke2016 | India | N | Flexibility training | NDI | 3 | 4 | 1 | 0.61 |
| 48 | Phadke2016 | India | N | Control | NDI | NA | NA | NA | 0.57 |
| 49 | Deng 2025 | China | N | Mind-body exercise | NDI | 2 | 3 | 8 | 0.58 |
| 49 | Deng2025 | China | N | Control | NDI | NA | NA | NA | NA |
| 50 | Leungbootnak 2026 | Thailand | N | Coordination | NDI | 3 | 2 | 6 | 0.66 |
| 50 | Leungbootnak 2026 | Thailand | N | Rehabilitation training | NDI | 2 | 2 | 6 | 0.66 |
| 51 | Çınar 2026 | Turkey | N | Aerobic | NDI | 3 | 3 | 8 | 0.15 |
| 51 | Çınar 2026 | Turkey | N | Aerobic+Resistanceke | NDI | 4 | 3 | 8 | 0.4 |

**Supplement 4: Risk of Bias in Individual Studies**

| cov_id | studyID | Bias arising from the randomization process | Bias due to deviations from intended interventions | Bias due to missing outcome data | Bias in measurement of the outcome | Bias in selection of the reported result | Overall bias |
| --- | --- | --- | --- | --- | --- | --- | --- |
| 1 | Yun2015 | Low risk | Some concerns | Low risk | Low risk | Low risk | Unclear |
| 2 | Michalsen2012 | Low risk | Low risk | Low risk | Low risk | Low risk | Low |
| 3 | Trott2009 | Low risk | Some concerns | Low risk | Low risk | Low risk | Unclear |
| 3 | Trott2009 | Low risk | Low risk | Some concerns | Some concerns | Some concerns | Unclear |
| 4 | Kang2021 | Some concerns | Some concerns | Some concerns | High risk | Low risk | High |
| 5 | Tunwattanapong2016 | Some concerns | Some concerns | Low risk | Some concerns | Low risk | Unclear |
| 6 | Picak2023 | Some concerns | Some concerns | High risk | High risk | High risk | High |
| 7 | Ris2016 | Some concerns | Some concerns | Low risk | Some concerns | Low risk | Unclear |
| 8 | Rendant2011 | Some concerns | Some concerns | Low risk | Some concerns | Low risk | Unclear |
| 9 | Park2015 | Some concerns | Some concerns | Some concerns | Some concerns | Some concerns | Unclear |
| 10 | Nandini2018 | Some concerns | Some concerns | Low risk | Some concerns | Low risk | Unclear |
| 11 | Ma2011 | Some concerns | Some concerns | Low risk | Low risk | Low risk | Unclear |
| 12 | Lytras2020 | Some concerns | Some concerns | Low risk | Some concerns | Low risk | Unclear |
| 13 | Li2017 | Some concerns | Low risk | Low risk | Low risk | Low risk | Unclear |
| 14 | Letafatkar2019 | Some concerns | Some concerns | Low risk | Low risk | Low risk | Unclear |
| 15 | Lee2016 | Low risk | Low risk | Low risk | Low risk | Low risk | Low |
| 16 | Lansinger2007 | Low risk | Some concerns | Low risk | Low risk | Low risk | Unclear |
| 17 | Kim2016 | Low risk | Low risk | Some concerns | Some concerns | Some concerns | Unclear |
| 18 | Khosrokiani2017 | Some concerns | Some concerns | Some concerns | High risk | Low risk | High |
| 19 | Karlsson2014 | Low risk | Some concerns | Low risk | Some concerns | Low risk | Unclear |
| 20 | Gupta2013 | Low risk | Some concerns | High risk | High risk | High risk | High |
| 21 | Lee2016_2 | Low risk | Some concerns | Low risk | Some concerns | Low risk | Unclear |
| 22 | Javdaneh2020 | Low risk | Some concerns | Low risk | Some concerns | Low risk | Unclear |
| 23 | Javdaneh2021 | Low risk | Some concerns | Some concerns | Some concerns | Some concerns | Unclear |
| 24 | Hakkinen2008 | Low risk | Some concerns | Low risk | Some concerns | Low risk | Unclear |
| 25 | Groisman2019 | Low risk | Some concerns | Low risk | Low risk | Low risk | Unclear |
| 26 | GRIFFITHS2009 | Low risk | Some concerns | Low risk | Some concerns | Low risk | Unclear |
| 27 | ULUG2018 | Low risk | Low risk | Low risk | Low risk | Low risk | Low |
| 28 | Ghaderi2016 | Low risk | Some concerns | Low risk | Low risk | Low risk | Unclear |
| 29 | Kjellman2002 | Low risk | Low risk | Low risk | Low risk | Low risk | Low |
| 30 | BoriSut2013 | Low risk | Some concerns | Low risk | Low risk | Low risk | Unclear |
| 30 | BoriSut2013 | Low risk | Low risk | Some concerns | Some concerns | Some concerns | Unclear |
| 31 | Chiu2005 | Some concerns | Some concerns | Some concerns | High risk | Low risk | High |
| 32 | Cramer2013 | Low risk | Some concerns | Low risk | Some concerns | Low risk | Unclear |
| 33 | Cazotti2018 | Low risk | Some concerns | High risk | High risk | High risk | High |
| 34 | Dunleavy2015 | Low risk | Some concerns | Low risk | Some concerns | Low risk | Unclear |
| 35 | Dusunceli2009 | Low risk | Some concerns | Low risk | Some concerns | Low risk | Unclear |
| 36 | Cheng2022 | Low risk | Some concerns | Some concerns | Some concerns | Some concerns | Unclear |
| 37 | Falla2013 | Low risk | Some concerns | Low risk | Some concerns | Low risk | Unclear |
| 38 | Evans2012 | Low risk | Some concerns | Low risk | Low risk | Low risk | Unclear |
| 39 | Bernal-Utrera2020 | Low risk | Some concerns | Low risk | Some concerns | Low risk | Unclear |
| 40 | Galindez-Ibarbengoetxea2017 | Low risk | Low risk | Low risk | Low risk | Low risk | Low |
| 41 | Bharti2024 | Low risk | Some concerns | Low risk | Low risk | Low risk | Unclear |
| 42 | Kuptniratsaikula2023 | Low risk | Some concerns | Low risk | Some concerns | Low risk | Unclear |
| 43 | Ahi2023 | Low risk | Low risk | Low risk | Low risk | Low risk | Low |
| 44 | Nejati2020 | Low risk | Some concerns | Low risk | Low risk | Low risk | Unclear |
| 45 | Ei-gendy2019 | Low risk | Some concerns | Low risk | Some concerns | Low risk | Unclear |
| 46 | Khan2022 | Low risk | Low risk | Low risk | Low risk | Low risk | Low |
| 47 | Kashyap2018 | Low risk | Some concerns | Low risk | Some concerns | Low risk | Unclear |
| 48 | Phadke2016 | Low risk | Low risk | Low risk | Low risk | Low risk | Low |
| 49 | Deng2025 | Low risk | Some concerns | Low risk | Low risk | Low risk | Unclear |
| 50 | Leungbootnak 2026 | Low risk | Some concerns | Low risk | Some concerns | Low risk | Unclear |
| 51 | Çınar 2026 | Low risk | Some concerns | Low risk | Low risk | Low risk | Unclear |

## Supplement 5: The details of GRADE

|  |  | | | | | | **Certainty** |
| --- | --- | --- | --- | --- | --- | --- | --- |
| **Total studies** | **Study design** | **Risk of bias** | **Inconsistency** | **Indirectness** | **Imprecision** | **Other considerations** |  |
| Aerobic | RCT | serious | not serious | not serious | seriousa | none | ⊕⊕OO  Low |
| Flexibility | RCT | not serious | not serious | not serious | seriousa | none | ⨁⨁⨁◯  Moderatea |
| Aerobic+Resistance | RCT | Serious | not serious | not serious | seriousa | none | ⊕⊕OO  Low |
| Coordination | RCT | not serious | not serious | not serious | seriousa | none | ⊕⊕OO  Low |
| Mind-body exercise | RCT | serious | not serious | not serious | seriousa | none | ⊕⊕OO  Low |
| Rehabilitation | RCT | serious | not serious | not serious | seriousa | none | ⊕⊕OO  Low |
| Resistance | RCT | serious | not serious | not serious | seriousa | none | ⊕⊕OO  Low |
| Control | RCT | serious | not serious | not serious | seriousa | none | ⊕⊕OO  Low |
| Resistance+coordination | RCT | serious | serious | serious | seriousa | none | OOOO  Very Low |

## Supplenment 6: Node-splitting analysis of inconsistency

| Comparison | NMA | Direct | Indirect | Diff | z | p |
| --- | --- | --- | --- | --- | --- | --- |
| **Aerobic : Control** | –1.05 | –0.99 | –1.15 | 0.16 | 0.14 | 0.889 |
| **Aerobic_Resistance : Control** | –1.00 | –0.91 | –1.21 | 0.30 | 0.16 | 0.873 |
| **Coordination : Control** | –1.35 | –1.20 | –2.50 | 1.30 | 0.55 | 0.582 |
| **Flexibility_training : Control** | –0.30 | –0.26 | –0.40 | 0.14 | 0.07 | 0.944 |
| **Mindbody_exercise : Control** | –0.75 | –0.60 | –1.85 | 1.25 | 1.45 | 0.147 |

**Supplement 7: League tables**

|  | **Mind-body** | **Resistance** | **Flexibility** | **Coordination** | **Aerobic + Resistance** | **Aerobic** | **Rehabilitation** | **Resistance + Coordination** | **CG** |
| --- | --- | --- | --- | --- | --- | --- | --- | --- | --- |
| Mind-body | — |  |  |  |  |  |  |  |  |
| Resistance | -0.26 (-0.30, -0.22) | — |  |  |  |  |  |  |  |
| Flexibility | -0.11 (-0.78, 0.54) | 0.29 (-0.39, 0.98) | — |  |  |  |  |  |  |
| Coordination | -0.58 (-1.69, 0.50) | -0.17 (-1.29, 0.93) | -0.52 (-0.91, -0.18) | — |  |  |  |  |  |
| Aerobic + Resistance | 0.62 (-0.38, 1.59) | 0.12 (-0.16, 0.40) | 0.74 (-0.29, 1.75) | 0.05 (-0.95, 1.06) | — |  |  |  |  |
| Aerobic | -0.47 (-1.90, 0.94) | 0.10 (-0.16, 0.36) | -0.35 (-1.79, 1.09) | 0.11 (-1.58, 1.79) | -0.55 (-1.00, 0.20) | — |  |  |  |
| Rehabilitation | 0.01 (-0.61, 0.59) | 0.41 (-0.23, 1.05) | 0.12 (-0.55, 0.77) | 0.58 (-0.21, 0.90) | -0.62 (-1.60, 0.36) | -0.22 (-0.42, -0.02) | — |  |  |
| Resistance + Coordination | -0.17 (-0.57, 0.24) | -0.15 (-0.61, 0.3) | -0.13 (-0.57, 0.31) | -0.16 (-0.6, 0.28) | -0.08 (-0.59, 0.42) | -0.02 (-0.51, 0.46) | 0.00 (-0.51, 0.51) | — |  |
| CG | -0.363 (-0.516, -0.151) | -0.359 (-0.611, -0.102) | -0.352 (-0.623, -0.078) | -0.328 (-0.579, -0.076) | -0.282 (-0.631, 0.075) | -0.220 (-0.575, 0.075) | -0.201 (-0.490, 0.235) | -0.198 (-0.510, 0.212) | — |


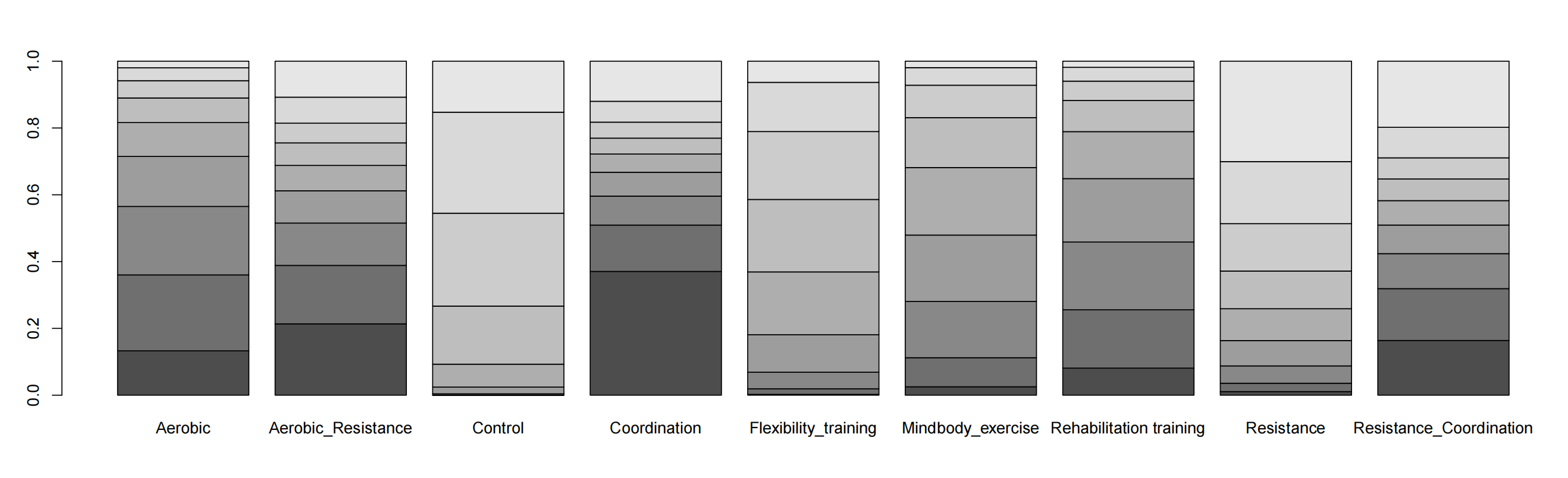


**Figure S1 SUCRA Ranking**

**Supplement 8: Funnel plot**


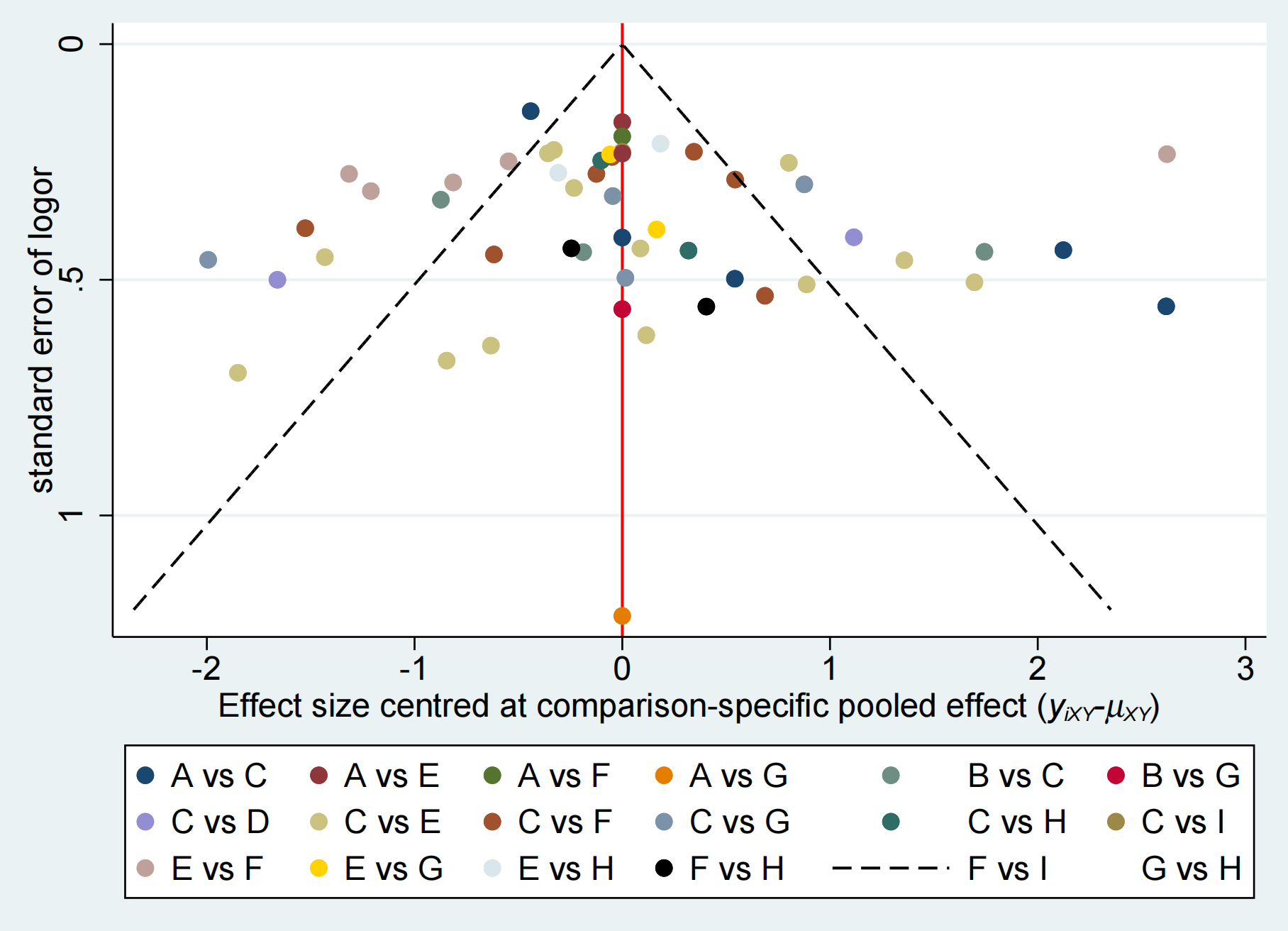


**Figure S2 Funnel plots**

#

# Supplement 9: Dose-response network meta-analyses


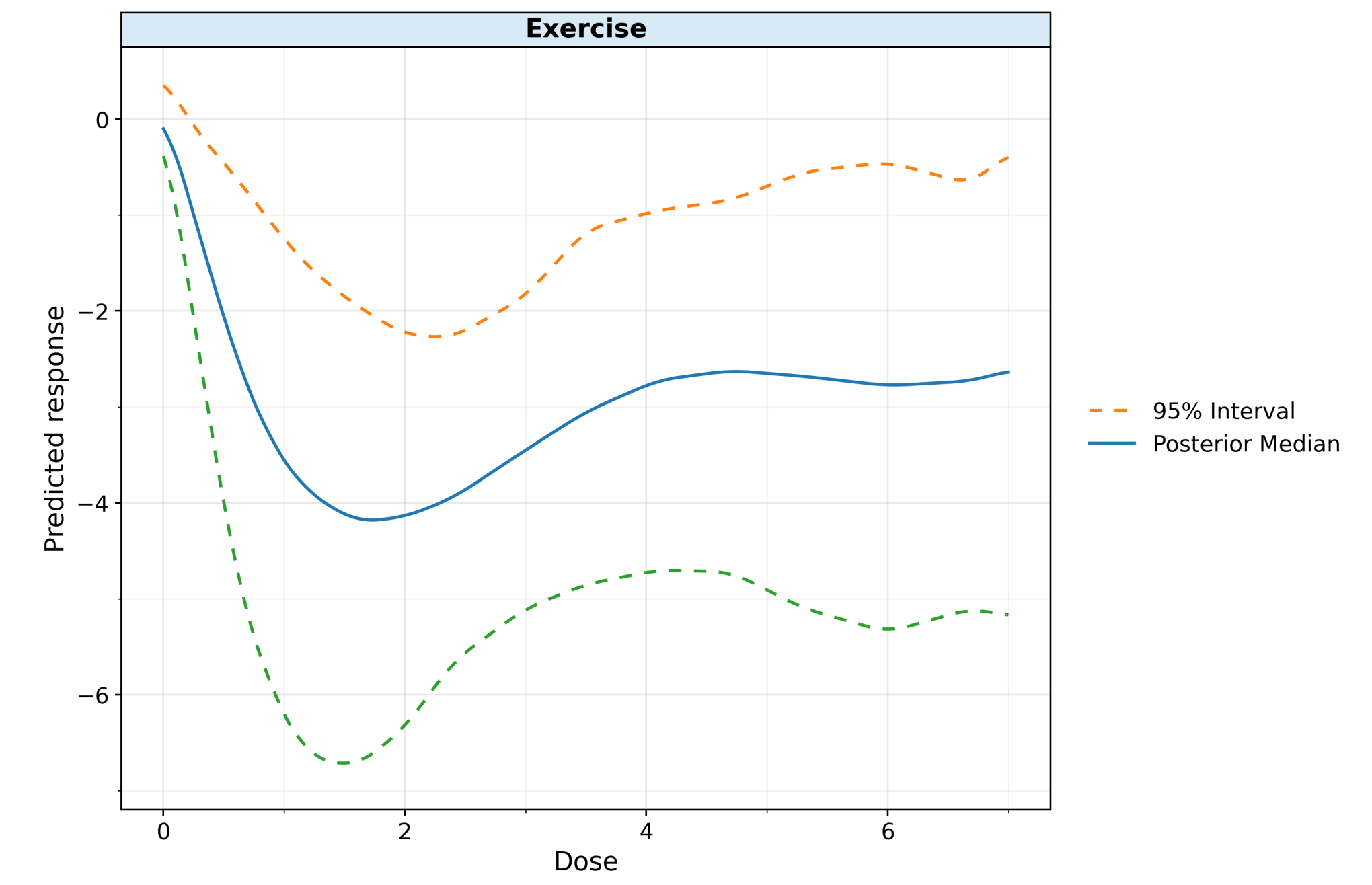
**Figure S3.** **Prediction dose-response relationship at exercise frequency.**

## Supplement 10:. Network meta-regression

Network meta-regression analyses of depression outcomes were performed to investigate potential moderating variables and the degree of model fit. The network meta-regression model was fitted in a Bayesian framework using the Markov chain Monte Carlo (MCMC) method in the R statistical package “Gemtc”. Four chains were run using a non-informative prior. The number of iterations for each chain was 25,000, with the first 5,000 iterations discarded.

**Table S5. Model fit summaries for univariate network meta-regression.**

| **Covariate** | **(92 data points)** | | | |
| --- | --- | --- | --- | --- |
|  | **DIC** | **pD** | **Residual**  **Deviance** | **Tau (SD)** |
| Unadjusted | 180.3 | 83.5 | 96.8 | 0.63 (0.45, 0.87) |
| Age | 180.2 | 83.7 | 96.4 | 0.61 ( 0.39,0.66) |
| Intervention  duration (weeks) | 178.5 | 83.5 | 96.3 | 0.65 (0.50,0.86) |
| Intervention  frequency (weekly) | 178.8 | 83.8 | 96.6 | 0.60 (0.46,0.81) |
| Publication years | 179.7 | 83.6 | 96.9 | 0.62 (0.44, 0.87) |
| Intervention timing | 178.6 | 83.4 | 96.1 | 0.63 (0.42, 0.87) |
| Study population | 177.8 | 83.1 | 95.8 | 0.60 (0.41, 0.82) |

***Note:*** CrI, credible interval. BMI, body mass index. DIC, deviance information criterion. SD, standard deviation.

**Reference**

1.Yun S. 2015. The effect of neurac training in patients with chronic neck pain. Journal of Physical Therapy Science 27:1303-1307.

2.Michalsen A. 2012. Yoga for Chronic Neck Pain: A Pilot Randomized Controlled Clinical Trial. The Journal of Pain 13:1122-1130.

3.von Trott P, Wiedemann AM, Lüdtke R, Reißhauer A, Willich SN, and Witt CM. 2009. Qigong and Exercise Therapy for Elderly Patients With Chronic Neck Pain (QIBANE): A Randomized Controlled Study. The Journal of Pain 10:501-508. 10.1016/j.jpain.2008.11.004

4.Kang N-Y, Im S-C, and Kim K. 2021. Effects of a combination of scapular stabilization and thoracic extension exercises for office workers with forward head posture on the craniovertebral angle, respiration, pain, and disability: A randomized-controlled trial. Turkish Journal of Physical Medicine and Rehabilitation 67:291-299. 10.5606/tftrd.2021.6397

5.Tunwattanapong P, Kongkasuwan R, and Kuptniratsaikul V. 2015. The effectiveness of a neck and shoulder stretching exercise program among office workers with neck pain: a randomized controlled trial. Clinical Rehabilitation 30:64-72. 10.1177/0269215515575747

6.Sahiner Picak G, and Yesilyaprak SS. 2022. Effects of clinical pilates exercises in patients with chronic nonspecific neck pain: a randomized clinical trial. Irish Journal of Medical Science (1971 -) 192:1205-1214. 10.1007/s11845-022-03101-y

7.Ris I, Søgaard K, Gram B, Agerbo K, Boyle E, and Juul-Kristensen B. 2016. Does a combination of physical training, specific exercises and pain education improve health-related quality of life in patients with chronic neck pain? A randomised control trial with a 4-month follow up. Manual Therapy 26:132-140. 10.1016/j.math.2016.08.004

8.Rendant D, Pach D, Lüdtke R, Reisshauer A, Mietzner A, Willich SN, and Witt CM. 2011. Qigong Versus Exercise Versus No Therapy for Patients With Chronic Neck Pain. Spine 36:419-427. 10.1097/BRS.0b013e3181d51fca

9.Park SD. 2015. Clinical feasibility of cervical exercise to improve neck pain, body function, and psychosocial factors in patients with post-traumatic stress disorder: a randomized controlled trial. Journal of Physical Therapy Science 27:1369-1371.

10.Nandini B, Mooventhan A, and Manjunath NK. 2018. Add-on Effect Of Hot Sand Fomentation To Yoga On Pain, Disability, And Quality Of Life In Chronic Neck Pain Patients. Explore 14:373-378. 10.1016/j.explore.2018.01.002

11.Ma C, Szeto GP, Yan T, Wu S, Lin C, and Li L. 2011. Comparing Biofeedback With Active Exercise and Passive Treatment for the Management of Work-Related Neck and Shoulder Pain: A Randomized Controlled Trial. Archives of Physical Medicine and Rehabilitation 92:849-858. 10.1016/j.apmr.2010.12.037

12.Lytras DE, Sykaras EI, Christoulas KI, Myrogiannis IS, and Kellis E. 2020. Effects of Exercise and an Integrated Neuromuscular Inhibition Technique Program in the Management of Chronic Mechanical Neck Pain: A Randomized Controlled Trial. Journal of Manipulative and Physiological Therapeutics 43:100-113. 10.1016/j.jmpt.2019.03.011

13.Li X, Lin C, Liu C, Ke S, Wan Q, Luo H, Huang Z, Xin W, Ma C, and Wu S. 2017. Comparison of the effectiveness of resistance training in women with chronic computer-related neck pain: a randomized controlled study. International Archives of Occupational and Environmental Health 90:673-683. 10.1007/s00420-017-1230-2

14.Letafatkar A, Rabiei P, Alamooti G, Bertozzi L, Farivar N, and Afshari M. 2019. Effect of therapeutic exercise routine on pain, disability, posture, and health status in dentists with chronic neck pain: a randomized controlled trial. International Archives of Occupational and Environmental Health 93:281-290. 10.1007/s00420-019-01480-x

15.Lee K-w. 2016a. Effect of thoracic manipulation and deep craniocervical flexor training on pain, mobility, strength, and disability of the neck of patients with chronic nonspecific neck pain: a randomized clinical trial. The Journal of Physical Therapy Science 28:175-180.

16.Lee S-M. 2016b. Clinical effectiveness of a Pilates treatment for forward head posture. The Journal of Physical Therapy Science 28:2009-2013.

17.Lansinger B, Carlsson JY, Kreuter M, and Taft C. 2013. Health-related quality of life in persons with long-term neck pain after treatment with qigong and exercise therapy respectively. European Journal of Physiotherapy 15:111-117. 10.3109/21679169.2013.805816

18.Kim JY. 2016. Clinical effects of deep cervical flexor muscle activation in patients with chronic neck pain. The Journal of Physical Therapy Science 28:269-273.

19.Khosrokiani Z, Letafatkar A, and Sokhanguei Y. 2018. Long-term effect of direction-movement control training on female patients with chronic neck pain. Journal of Bodywork and Movement Therapies 22:217-224. 10.1016/j.jbmt.2017.06.004

20.Karlsson L. 2014. Evaluation of pain and function after two home exercise programs in a clinical trial on women with chronic neck pain - with special emphasises on completers and responders. BMC Musculoskeletal Disorders 15:1-13.

21.Gupta BD. 2013. Effect of Deep Cervical Flexor Training vs. Conventional Isometric Training on Forward Head Posture, Pain, Neck Disability Index In Dentists Suffering from Chronic Neck Pain. Journal of Clinical and Diagnostic Research. 10.7860/jcdr/2013/6072.3487

22.Javdaneh N, Letafatkar A, Shojaedin S, and Hadadnezhad M. 2020. Scapular exercise combined with cognitive functional therapy is more effective at reducing chronic neck pain and kinesiophobia than scapular exercise alone: a randomized controlled trial. Clinical Rehabilitation 34:1485-1496. 10.1177/0269215520941910

23.Javdaneh N, Molayei F, and Kamranifraz N. 2021a. Effect of adding motor imagery training to neck stabilization exercises on pain, disability and kinesiophobia in patients with chronic neck pain. Complementary Therapies in Clinical Practice 42. 10.1016/j.ctcp.2020.101263

24.Hakkinen A. 2008. Strength training and stretching versus stretching only in the treatment of patients with chronic neck pain: a randomized one-year follow-up study. Clinical Rehabilitation 22:592-600.

25.Groisman S, Malysz T, de Souza da Silva L, Rocha Ribeiro Sanches T, Camargo Bragante K, Locatelli F, Pontel Vigolo C, Vaccari S, Homercher Rosa Francisco C, Monteiro Steigleder S, and Jotz GP. 2020. Osteopathic manipulative treatment combined with exercise improves pain and disability in individuals with non-specific chronic neck pain: A pragmatic randomized controlled trial. Journal of Bodywork and Movement Therapies 24:189-195. 10.1016/j.jbmt.2019.11.002

26.Griffiths C, Dziedzic K, Waterfield J, and Sim J. 2009. Effectiveness of Specific Neck Stabilization Exercises or a General Neck Exercise Program for Chronic Neck Disorders: A Randomized Controlled Trial. The Journal of Rheumatology 36:390-397. 10.3899/jrheum.080376

27.Uluğ N, Yılmaz, Kara M, and Özçakar L. 2018. Effects of Pilates and yoga in patients with chronic neck pain: A sonographic study. Journal of Rehabilitation Medicine 50:80-85. 10.2340/16501977-2288

28.Ghaderi F, Jafarabadi MA, and Javanshir K. 2017. The clinical and EMG assessment of the effects of stabilization exercise on nonspecific chronic neck pain: A randomized controlled trial. Journal of Back and Musculoskeletal Rehabilitation 30:211-219. 10.3233/bmr-160735

29.Kjellman G. 2002. A RANDOMIZED CLINICAL TRIAL COMPARING GENERAL EXERCISE, MCKENZIE TREATMENT AND A CONTROL GROUP IN PATIENTS WITH NECK PAIN. Journal of Rehabilitation Medicine 34:183-190.

30.BoriSut. S. 2013. Effects of Strength and Endurance Training of Superficial and Deep Neck Muscles on Muscle Activities and Pain Levels of Females with Chronic Neck Pain. Journal of Physical Therapy Science 225:1157-1162.

31.Chiu TT. 2005. A randomized clinical trial of TENS and exercise for patients with chronic neck pain. Clinical Rehabilitation 19:850-860.

32.Cramer H. 2013. Randomized-controlled Trial Comparing Yoga and Home-based Exercise for Chronic Neck Pain. CLINICAL JOURNAL OF PAIN 29:216-223.

33.de Araujo Cazotti L, Jones A, Roger-Silva D, Ribeiro LHC, and Natour J. 2018. Effectiveness of the Pilates Method in the Treatment of Chronic Mechanical Neck Pain: A Randomized Controlled Trial. Archives of Physical Medicine and Rehabilitation 99:1740-1746. 10.1016/j.apmr.2018.04.018

34.Dunleavy K, Kava K, Goldberg A, Malek MH, Talley SA, Tutag-Lehr V, and Hildreth J. 2016. Comparative effectiveness of Pilates and yoga group exercise interventions for chronic mechanical neck pain: quasi-randomised parallel controlled study. Physiotherapy 102:236-242. 10.1016/j.physio.2015.06.002

35.Dusunceli Y, Ozturk C, Atamaz F, Hepguler S, and Durmaz B. 2009. Efficacy of neck stabilization exercises for neck pain: A randomized controlled study. Journal of Rehabilitation Medicine 41:626-631. 10.2340/16501977-0392

36.Cheng Z-j, Zhang S-p, Gu Y-j, Chen Z-y, Xie F-f, Guan C, Fang M, and Yao F. 2022. Effectiveness of Tuina Therapy Combined With Yijinjing Exercise in the Treatment of Nonspecific Chronic Neck Pain. JAMA Network Open 5. 10.1001/jamanetworkopen.2022.46538

37.Falla D, Lindstrøm R, Rechter L, Boudreau S, and Petzke F. 2013. Effectiveness of an 8‐week exercise programme on pain and specificity of neck muscle activity in patients with chronic neck pain: A randomized controlled study. European Journal of Pain 17:1517-1528. 10.1002/j.1532-2149.2013.00321.x

38.Evans R, Bronfort G, Schulz C, Maiers M, Bracha Y, Svendsen K, Grimm R, Garvey T, and Transfeldt E. 2012. Supervised Exercise With and Without Spinal Manipulation Performs Similarly and Better Than Home Exercise for Chronic Neck Pain. Spine 37:903-914. 10.1097/BRS.0b013e31823b3bdf

39.Bernal-Utrera C, Gonzalez-Gerez JJ, Anarte-Lazo E, and Rodriguez-Blanco C. 2020. Manual therapy versus therapeutic exercise in non-specific chronic neck pain: a randomized controlled trial. Trials 21. 10.1186/s13063-020-04610-w

40.Galindez-Ibarbengoetxea X, Setuain I, Ramírez-Velez R, Andersen LL, González-Izal M, Jauregi A, and Izquierdo M. 2018. Short-term effects of manipulative treatment versus a therapeutic home exercise protocol for chronic cervical pain: A randomized clinical trial. Journal of Back and Musculoskeletal Rehabilitation 31:133-145. 10.3233/bmr-169723

41.Bharti N, Ahmed H, Hasan S, Iqbal A, Uddin S, Ahamed WM, Ahmad F, Mujtaba MA, Alghadir AH, and Valeriani M. 2024. Efficacy of Scapular Functional and Cervical Isometric Exercises in the Management of Chronic Mechanical Neck Pain: A Randomized Comparative Trial. Pain Research and Management 2024. 10.1155/prm/5873384

42.Kuptniratsaikula V. 2023. Pain reduction, physical performance, and psychological status compared between Hatha yoga and stretching exercise to treat sedentary office workers with mild/moderate neck/shoulder pain: A randomized controlled non-inferiority trial*. Complementary Therapies in Medicine 79:1-11.

43.Ahi ED, and Sirzai H. 2022. Comparison of the effectiveness of dry needling and high-intensity laser therapy in the treatment of myofascial pain syndrome: a randomized single-blind controlled study. Lasers in Medical Science 38. 10.1007/s10103-022-03687-w

44.Nejati P, Mousavi R, and Angoorani H. 2020. Acupuncture is as Effective as Exercise for Improvement of Chronic Neck Pain: A Randomized Clinical Trial. Shiraz E-Medical Journal 22. 10.5812/semj.97497

45.El-Gendy MH, Lasheen YR, and Rezkalla WKS. 2019. Multimodal approach of electrotherapy versus myofascial release in patients with chronic mechanical neck pain: a randomized controlled trial. Physiotherapy Quarterly 27:6-12. 10.5114/pq.2019.87735

46.Khan ZK, Ahmed SI, Baig AAM, and Farooqui WA. 2022. Effect of post-isometric relaxation versus myofascial release therapy on pain, functional disability, rom and qol in the management of non-specific neck pain: a randomized controlled trial. BMC Musculoskeletal Disorders 23. 10.1186/s12891-022-05516-1

47.Kashyap R, Iqbal A, and Alghadir AH. 2018. Controlled intervention to compare the efficacies of manual pressure release and the muscle energy technique for treating mechanical neck pain due to upper trapezius trigger points. Journal of Pain Research Volume 11:3151-3160. 10.2147/jpr.S172711

48.Phadke A, Bedekar N, Shyam A, and Sancheti P. 2016. Effect of muscle energy technique and static stretching on pain and functional disability in patients with mechanical neck pain: A randomized controlled trial. Hong Kong Physiotherapy Journal 35:5-11. 10.1016/j.hkpj.2015.12.002

49. Deng K, Zhou Y, Qian J, Wang L, Yu F, and Wang B. 2025. Effects of Tai Chi combined with intermediate frequency therapy on patients with chronic nonspecific neck pain: a randomized controlled trial. Frontiers in Pain Research 6:1700212. 10.3389/fpain.2025.1700212

50. Leungbootnak A, Puntumetakul R, Chatprem T, Hunsawong T, Wanpen S, Sae-Jung S, and Boucaut R. 2026. Effects of specific extensor exercise on balance control in patients with neck pain; a randomized controlled trial. BMC Complementary Medicine and Therapies. 10.1186/s12906-026-05366-7

51. Ayyıldız Çınar S, Demirel A, Şahiner ML, Öge HK, and Bilgin S. 2026. A Three-Arm Randomized Trial of Aerobic Training Versus High-Intensity Interval Training on Clinical and Functional Outcomes in Patients with Chronic Neck Pain. Nigerian Journal of Clinical Practice 29:113-126. 10.4103/njcp.njcp_476_25
