# Supplementary material for: Exercise modalities associated with reduced functional disability in nonspecific neck pain: a network meta-analysis and exploratory dose-response analysis
Source: PeerJ. 2026 Jul 23;14:e21534. doi: 10.7717/peerj.21534 (PMC13401843; doi:10.7717/peerj.21534)
Supplement: Supplemental Information 3 [file peerj-14-21534-s003.docx]

工作簿10086.csv Workbook10086.csv

网状meta分析课件（0） Network meta-analysis courseware (0)

第七章 Chapter 7

R的GEMTC做回归分析 Regression analysis using GEMTC in R

髋关节 Hip joint

数据分析 Data analysis

贝叶斯分析 Bayesian analysis

回归分析 Regression analysis

回归_fatigue_week.csv Regression_fatigue_week.csv

糖尿病数据分析 Diabetes data analysis

貝葉斯框架下的網狀meta分析 Network meta-analysis under a Bayesian framework

查看數據 View/check the data

網絡圖 Network plot

建立模型 Build the model

結果 Results

收斂圖 Convergence plot

點不一致性檢驗 Node-splitting inconsistency test

查看結果 View/check the results

查看节点劈裂法的结果 View the results of the node-splitting method

點不一致性森林圖 Node-splitting inconsistency forest plot

異質性檢驗 Heterogeneity test

排序 Ranking

聯賽圖 League table

森林圖 Forest plot

網狀meta回歸：轉化數據 Network meta-regression: transform data

回歸 Regression

建立回歸模型 Build the regression model

回歸結果 Regression results

森林圖調整 Forest plot adjustment

取數據前兩列作為文本 Use the first two columns of the data as text labels

v6列作為mean Use column V6 as the mean

v5列作為下線 Use column V5 as the lower limit

v7列作為上限 Use column V7 as the upper limit

森林圖的位置（第三列） Position of the forest plot (third column)

無效線為0 Set the null/no-effect line at 0

不要轉化成log值 Do not transform to log values

森林圖橫線以及點的顏色 Colors of the forest plot horizontal lines and points

置信區間線條的類型 Line type of the confidence interval

置信區間線條的寬度 Line width of the confidence interval

森林图可信区间两端添加小竖线（TRUE） Add small vertical lines at both ends of the forest plot confidence interval (TRUE)

設置森林圖可信區間兩段的小豎線的高度 Set the height of the small vertical lines at both ends of the forest plot confidence interval

设置表格中文本的格式：用gpar进行赋值，其中cex为文本字体大小，ticks为坐标轴大小，xlab为坐标轴文字字体大小。 Set the text format in the table: assign values using gpar; cex is the text font size, ticks is the axis tick-label size, and xlab is the axis-title font size.

label：表格主体文字的格式 label: format of the main text in the table

ticks：森林图下方的坐标轴的刻度文字格式 ticks: format of the tick labels on the axis below the forest plot

xlab：定义的x轴标题格式 xlab: format of the defined x-axis title

title：标题文字的格式 title: format of the title text

行的高度 Row height

列与列之间的间隙宽度 Gap width between columns

x轴的标题 Title of the x-axis

横坐标刻度根据需要可随意设置 The x-axis tick marks can be set as needed

每列文字的对齐方式。如：align=c("l","c","c") Alignment of the text in each column. For example: align=c("l","c","c")

左对齐 Left aligned

右对齐 Right aligned

居中对齐 Center aligned

森林图在表中的宽度 Width of the forest plot in the table

x軸線寬 Line width of the x-axis

點的形狀 Shape of the point markers

设定工作目录 Set the working directory

装载gemtc包，如果第一次用，需要先安装：install.packages("gemtc") Load the gemtc package; if using it for the first time, install it first with install.packages("gemtc")

把我们的数据文件导入到R里面 Import our data file into R

相当于数据的预处理 Equivalent to data preprocessing

网状图的输出 Output of the network plot

构建模型 Construct the model

迭代，产生结果 Run iterations and generate results

不一致性模型 最后分析 Inconsistency model; final analysis

与placebo相比较的森林图 Forest plot compared with placebo

收敛性诊断 Convergence diagnostics

输出轨迹图、密度图等 Output trace plots, density plots, etc.

排序概率 Ranking probabilities

计算SUCRA的函数 Function for calculating SUCRA

导出联赛表 Export the league table

将干预按照SUCRA大小进行排序的函数 Function for ranking interventions according to SUCRA values

到处按照SUCRA排序的联赛表 Export the league table sorted according to SUCRA

局部不一致性， 节点劈裂法 Local inconsistency: node-splitting method

异质性分析，谨慎使用 Heterogeneity analysis; use with caution

用ggplot包制作排序概率图 Use the ggplot package to create a rank-probability plot

制作累积排序图 Create a cumulative ranking plot

回归2 Regression 2

导入multinma包 Import the multinma package

多核并行计算 Multicore parallel computation

计算标准误 Calculate the standard error

设置网络属性 Set network attributes

输入af_net可查看设置好的网络属性： Enter af_net to view the configured network attributes:

绘制网状图 Draw the network plot

设定贝叶斯模型并运行 Specify and run the Bayesian model

选择随机效应模型，若选择固定则输入fixed Choose the random-effects model; enter fixed to choose the fixed-effect model

选择一致性模型，不一致模型则输入ume Choose the consistency model; enter ume for the inconsistency model

若想生成两两干预之间互相比较的结果，可以输入： To generate pairwise comparison results between interventions, enter:

若想生成预测区间，那么设置predictive_distribution参数，如下： To generate prediction intervals, set the predictive_distribution parameter as follows:

干预排名 Intervention ranking

若值越大越好，则改为FALSE If larger values are better, change this to FALSE

各个干预的排序概率 Ranking probabilities for each intervention

各个干预的累积排序概率 Cumulative ranking probabilities for each intervention

为了判断global inconsistency，我们需要比较一致性模型和不一致模型的DIC。 To assess global inconsistency, we need to compare the DIC of the consistency model and the inconsistency model.

之前我们做的一致性模型的DIC为： The DIC of the consistency model fitted earlier is:

接下来我们拟合不一致模型： Next, we fit the inconsistency model:

局部不一致分析（节点劈裂法） Local inconsistency analysis (node-splitting method)

还可以图片形式展示结果，输入： The results can also be displayed as a figure by entering:
